# An *In Vitro* Study on the Effects of Nisin on the Antibacterial Activities of 18 Antibiotics against *Enterococcus faecalis*

**DOI:** 10.1371/journal.pone.0089209

**Published:** 2014-02-20

**Authors:** Zhongchun Tong, Yuejiao Zhang, Junqi Ling, Jinglei Ma, Lijia Huang, Luodan Zhang

**Affiliations:** 1 Department of Operative Dentistry and Endodontics, Guanghua School of Stomatology, Sun Yat-sen University, Guangzhou, Guangdong, China; 2 Guangdong Provincial Key Laboratory of Stomatology, Sun Yat-sen University, Guangzhou, Guangdong, China; University of Kansas, United States of America

## Abstract

*Enterococcus faecalis* rank among the leading causes of nosocomial infections worldwide and possesses both intrinsic and acquired resistance to a variety of antibiotics. Development of new antibiotics is limited, and pathogens continually generate new antibiotic resistance. Many researchers aim to identify strategies to effectively kill this drug-resistant pathogen. Here, we evaluated the effect of the antimicrobial peptide nisin on the antibacterial activities of 18 antibiotics against *E. faecalis*. The MIC and MBC results showed that the antibacterial activities of 18 antibiotics against *E. faecalis* OG1RF, ATCC 29212, and strain E were significantly improved in the presence of 200 U/ml nisin. Statistically significant differences were observed between the results with and without 200 U/ml nisin at the same concentrations of penicillin or chloramphenicol (p<0.05). The checkerboard assay showed that the combination of nisin and penicillin or chloramphenicol had a synergetic effect against the three tested *E. faecalis* strains. The transmission electron microscope images showed that *E. faecalis* was not obviously destroyed by penicillin or chloramphenicol alone but was severely disrupted by either antibiotic in combination with nisin. Furthermore, assessing biofilms by a confocal laser scanning microscope showed that penicillin, ciprofloxacin, and chloramphenicol all showed stronger antibiofilm actions in combination with nisin than when these antibiotics were administered alone. Therefore, nisin can significantly improve the antibacterial and antibiofilm activities of many antibiotics, and certain antibiotics in combination with nisin have considerable potential for use as inhibitors of this drug-resistant pathogen.

## Introduction

Antibiotics have saved the lives of millions of people, greatly improving human and animal health in the twentieth century. However, bacterial pathogens commonly develop resistance to many antibiotics due to the extensive use of these antibiotics for human and animal health. Hundreds of thousands of deaths occur annually due to antibiotic treatment failures [1]. At present, the routine approach to addressing this crisis is to develop novel antibiotics. However, novel antibiotics are limited, and pathogens will gradually evolve resistance to these novel antibiotics [2]. Based on the inevitable trend towards bacterial resistance, it is necessary to explore new treatment strategies for effectively killing and eliminating bacterial pathogens. Limiting the evolution of bacterial resistance and using new and existing antibiotics may constitute a new strategy for antibacterial therapy.

Antimicrobial peptides (AMP) have been studied for the development of new antibacterial drugs due to their high antibacterial activity and low drug resistance [3], [4]. Although AMPs represent a potentially new source of antimicrobials for the treatment of various bacterial infections, conventional antibiotics remain a primary resource for antibacterial therapy and cannot be fully replaced at present. Therefore, combining conventional antibiotics and AMPs can prolong the life spans of many antibiotics. Nisin, an AMP from *Lactococcus lactis*, consists of 34 amino acid residues and is minimally toxic, odorless, colorless, and tasteless [5]. Nisin possesses high antimicrobial activity against a wide range of Gram-positive bacteria, even against some antibiotic-resistant pathogens [5], [6]. Some papers have reported the anti-pathogen activity of antibiotics in combination with nisin [7], [8], [9], [10], [11].

Antibiotic-resistant enterococci are one of major causes of hospital-acquired infections, as enterococci are common residents in the gastrointestinal tracts of a wide range of humans and animals. In *Enterococcus spp*, *Enterococcus faecalis* ranks among the leading causes of nosocomial infections worldwide [12]. *E. faecalis* has both an intrinsic and acquired resistance to a variety of antibiotics, including vancomycin and linezolid, and it is difficult to kill with antibiotic therapy alone [13]. The present study evaluates the antibacterial activities of 18 conventional antibiotics in combination with nisin against three *E. faecalis* strains grown under routine culture conditions, as well as biofilms, thus exploring the feasibility of combinations of nisin and antibiotics against drug-resistant pathogens.

## Materials and Methods

### Bacteria

This study used *E. faecalis* ATCC 29212, *E. faecalis* OG1RF, and *E. faecalis* strain E (A strain isolated from a root canal of a tooth with persistent apical periodontitis, exhibiting relatively high resistance, Tong *et al*. [14]). The three *E. faecalis* strains were routinely streaked on brain heart infusion agar (BHI; Difco Laboratories, Detroit, MI, USA) and cultured aerobically at 37°C for 24 h. A single bacterial colony was inoculated into 5 ml of BHI medium and grown to the exponential phase at an OD_600_ of 0.5.

### Preparation of the Antibiotics

Penicillin, ampicillin, gentamicin, kanamycin, roxithromycin, sulfapyridine, streptomycin, vancomycin, chloramphenicol, cefuroxime, cephazolin, ceftriaxone, cefepime, metronidazole, ciprofloxacin, polymyxin, imipenem, and linezolid were prepared at the concentration of 4,096 mg/L (all antibiotics were obtained from the First Affiliated Hospital, Sun Yat-sen University, Guangzhou, China). Nisin stock solution was obtained by dissolving 1 g of nisin powder (2.5% purity, 1000 U/mg, Sigma-Aldrich, St. Louis, MO) in 25 ml of dilute HCl (pH 2). All of the antimicrobials were filtered using a Millipore filter with a 0.22 µm pore size.

### The Determination of Minimum Inhibitory Concentration (MIC) and Minimum Bactericidal Concentration (MBC)

The MIC and MBC were determined by the microplate dilution method in Mueller-Hinton broth (MH, Becton, Dickinson & Co., Sparks, MD) with the addition of 5% lysed horse blood following the recommendations of the Clinical and Laboratory Standards Institute [15]. Briefly, the test antibiotics and nisin were diluted in 2-fold increments from 1∶1 to 1∶2,096 in MH broth. *E. faecalis* culture (OD_600_ of 0.5) was adjusted to 10^6^ CFU/ml with MH broth. The antibiotics and bacterial solutions were added in a 1∶1 ratio to 96-well microplates and then incubated at 37°C for 24 h. The MIC is defined as the lowest concentration of an antimicrobial agent at which the bacterial growth is completely inhibited [16]. A plate count of viable cells was performed to evaluate the MBC of the antimicrobials. Briefly, 10 µl of bacterial solution from each well that was considered the MIC was spread on the MH agar plates. The bacterial colonies were counted after the plates were incubated at 37°C for 24 h. The MBC was defined as the lowest concentration of an antibiotic that killed >99.9% of the total bacteria [17]. Furthermore, to evaluate the effect of nisin on the antibacterial activity of the test antibiotics, nisin solution was added to each well at 200 U/ml and the same determination of MIC and MBC was carried out. The assays were performed three times on different days.

### Checkerboard

A checkerboard assay was used to investigate the synergetic effects between nisin and penicillin or chloramphenicol. The procedure was referred to in a previous study [18]. Briefly, for the synergetic assay of nisin and penicillin, the rows of a 96-well microplate contained the same concentrations of nisin, diluted two-fold from 2000 to 31.25 U/ml along the y-axis. The column contained the same concentration of penicillin, diluted two-fold from 32 to 0.0625 mg/L along the x-axis. The combined effects of nisin and penicillin were captured by the fractional inhibitory concentration (FIC) index. The FIC index was calculated according to the equation: FIC index = FIC A+FIC B = (MIC of antimicrobial A in combination/MIC of A alone)+(MIC of antimicrobial B in combination/MIC of B alone). Synergism was defined as an FIC index ≤0.5; the additive effect as an FIC index of 0.5–4.0 and antagonism as an FIC index ≥4.0 [19]. The plates were subsequently incubated at 37°C for 24 h. The procedure was performed three times on different days. Similarly, the synergetic effect between nisin and chloramphenicol was evaluated by the same procedure.

### Bacterial Survival After Treatment with Four Antibiotics Alone or in Combination with Nisin

*E. faecalis* survival rates were evaluated after treatment with penicillin, vancomycin, chloramphenicol, and linezolid alone or in combination with nisin. In this assay, *E. faecalis* ATCC 29212 culture, nisin solution, and different concentrations of antibiotics were added into 96-well microplates. The resulting concentrations of cells and nisin were 5×10^7^ CFU/ml and 200 U/ml, respectively, and the four antibiotics were added to the wells according to the following concentration gradients: penicillin from 1028 to 2 mg/L; chloramphenicol from 1028 to 32 mg/L; vancomycin, from 1028 to 8 mg/L; linezolid, from 512 to 8 mg/L. Meanwhile, the microplates with the test antibiotics alone and *E. faecalis* solution, or with 200 U/ml nisin alone and *E. faecalis* solution, were referred to as controls. After the experimental and control microplates were incubated at 37°C for 24 h, the surviving bacteria in each well were counted on through a plate count of viable cells. The assays were carried out three times on different days.

### Transmission Electron Microscope

The morphological changes of *E. faecalis* were observed by transmission electron microscopy after treatment with the antibiotics in combination with nisin. Six groups of exponential phase *E. faecalis* ATCC 29212 were challenged by the following drugs for 12 h: 1024 mg/L penicillin (A); 1024 mg/L chloramphenicol (B); 2000 U/ml nisin (C); 1024 mg/L penicillin and 2000 U/ml nisin (D); 1024 mg/L chloramphenicol and 2000 U/ml nisin (E); and phosphate buffered saline (PBS) (Control). After treatment, sample *E. faecalis* cells were prepared according to the TEM analysis of the Dufour, *et al*. study [20]. In brief, the cell depositions were fixed with 3% glutaraldehyde, frozen, dehydrated with increasing concentrations of ethanol, and embedded in resin. The specimens were examined using a transmission electron microscope (TEM, Tecnai™ G2, FEI Company, American).

### Confocal Laser Scanning Microscope

The antibiofilm activities of penicillin, ciprofloxacin, and chloramphenicol in combination with nisin against *E. faecalis* were evaluated by a Live/Dead BacLight Bacterial Viability Kit (L13152; Molecular Probes, Invitrogen, Inc.,Eugene, OR, USA). For the *E. faecalis* biofilm, 1.98 ml of tryptic soy broth (TSB, BD DIFCO, Sparks MD, USA) supplemented with 1% glucose and 20 µl of an overnight culture of *E. faecalis* ATCC 29212 were added into the seven flat bottom plastic wells. Petri dishes with glass bottoms (D: 35 mm, Hangzhou Shengyou Biotechnology, China) were then incubated at 37°C for 24 h, and *E. faecalis* biofilms were generated on the surfaces of their glass bottoms. Subsequently, the seven biofilm groups were slightly washed twice with sterile PBS and then challenged with the following antimicrobials for 12 h: 512 mg/L penicillin (A); 512 mg/L ciprofloxacin (B); 512 mg/L chloramphenicol (C); 400 U/ml nisin (D); 512 mg/L penicillin and 400 U/ml nisin (E); 512 mg/L ciprofloxacin and 400 U/ml nisin (F); and 512 mg/L chloramphenicol and 400 U/ml nisin (G). The seven biofilm groups were stained with a mixture of 6 µM SYTO 9 stain and 30 µM PI at room temperature in the dark for 15 min, according to the specifications of the L13152 Kit. The stained specimens were then clamped and scanned using a Carl Zeiss confocal laser scanning microscope (CLSM) and ZEN software (ZEN 2010 light edition, Carl Zeiss MicroImaging, Inc., Thornwood, NY). SYTO 9 and PI were excited at 488 nm and 543 nm, respectively. Three-dimensional *E. faecalis* biofilms that formed after 12 h of treatment with 512 mg/L penicillin, 400 U/ml nisin, a combination of the two antimicrobials, and the controls without treatment were scanned along the Z axis.

### Statistical Analysis

Statistical analysis was performed using SPSS 18.0 software. Raw *E. faecali*s colony counts were transformed to log10 values to normalize the data. The student’s t test was used to compare the survival of *E. faecali*s after 12 h of treatment with each concentration of penicillin, vancomycin, chloramphenicol, or linezolid alone and in conjunction with nisin. A P value <0.05 was considered statistically significant.

## Results

### MIC, MBC and Checkerboard

The MICs of nisin against *E. faecali*s strains ATCC 29212, OG1RF, and strain E were 1,000 U/ml (1 mg/ml), and their MBCs were 2,000 U/ml (2 mg/ml). The resistances of the three strains of *E. faecalis* to 18 antibiotics alone or in combination with 200 U/ml nisin are shown in Table 1. Penicillin, ampicillin, vancomycin, ciprofloxacin, imipenem, and linezolid all exhibited higher antibacterial activity against the three *E. faecali*s strains than any of the other antibiotics used, and most of the MICs of the six antibiotics were less than or equal to 8 mg/L.

**Table 1:** The MIC and MBC of 18 antibiotics alone or in combination with 200/ml nisin against three *Enterococcus faecalis* strains.

Antibiotics (mg/L)	*Enterococcus faecalis* OG1RF				*Enterococcus faecalis* ATCC 29212				*Enterococcus faecalis* strain E			
	MIC	MBC	MIC*	MBC*	MIC	MBC	MIC*	MBC*	MIC	MBC	MIC*	MBC*
Penicillin	2	4	1	4	2	16	0.5	1	2	32	2	2
Ampicillin	4	32	1	2	4	16	0.5	4	2	4	2	4
Gentamicin	8	64	1	8	4	128	1	16	128	256	64	64
Kalamycin	16	64	0.5	8	512	1024	8	64	512	1024	128	128
Roxithromycin	8	256	1	4	64	64	1	4	>1024	>1024	16	64
Sulfapyridine	>1024	>1024	>1024	>1024	>1024	>1024	>1024	>1024	>1024	>1024	>1024	>1024
Streptomycin	64	256	4	32	16	512	2	4	256	1024	256	256
Vancomycin	4	128	1	16	4	256	0.5	8	8	>1024	4	8
Chloramphenicol	8	128	1	8	32	256	1	8	16	512	4	16
Cefuroxime sodium	512	1024	2	32	256	>1024	1	8	128	>1024	8	>1024
Cephazolin sodium	16	512	1	8	16	512	2	4	8	>1024	4	32
Ceftriaxone sodium	256	1024	4	32	64	>1024	1	16	512	>1024	64	512
Cefepime	64	256	0.25	32	64	1024	1	8	32	>1024	32	64
Metronidazol	>1024	>1024	>1024	>1024	>1024	>1024	>1024	>1024	>1024	>1024	>1024	>1024
Ciprofloxacin	2	8	0.25	2	1	16	0.5	2	16	512	0.5	2
Polymyxin	1024	>1024	256	>1024	512	>1024	128	>1024	>1024	>1024	>1024	>1024
Imipenem	2	8	0.5	1	1	4	0.5	2	4	16	4	8
Linezolid	2	32	0.125	16	8	32	0.5	4	8	128	1	2
nisin	1000	2000	–	–	1000	2000	–	–	1000	2000	–	–

“*” indicated the MIC and MBC of antibiotics in combination with 200 U/ml nisin against *E. faecalis*. 1000 mg/L nisin is equal to 1000 U/ml nisin.

Of the 18 tested antibiotics, 3, 5, and 9 antibiotics did not result in detectable MBCs against OG1RF, ATCC 29212, and strain E, respectively, and *E. faecali*s strains exhibited a high level of resistance to the antibiotics. Furthermore, for some antibiotics such as kanamycin and streptomycin, the MBC could only be detected at higher concentrations. However, in the assay of the antibiotics in combination with the antimicrobial peptide nisin, 200 U/ml nisin significantly improved the antibacterial and bactericidal activities of all of the tested antibiotics. The exceptions were sulfapyridine, metronidazol, and polymyxin, none of which had much of an antibacterial effect on *E. faecali*s. At the test concentrations, several antibiotics such as roxithromycin, streptomycin, cefuroxime, cephazolin, ceftriaxone, and cefepime did not exhibit good antibacterial activity against the test strain *E. faecalis*, but the MIC and MBC of the antibiotics were significantly decreased when combined with 200 U/ml nisin.

For the synergetic effect between nisin and penicillin, the FIC of nisin against the OG1RF, ATCC 29212, and strain E was 0.06, 0.125, and 0.03, respectively, and the FIC of penicillin against the three strains was 0.25. Thus, the total FICs of nisin and penicillin were 0.31, 0.375, and 0.28, respectively. Furthermore, in the synergetic effect between nisin and chloramphenicol, the FIC of nisin against the strain E, OR1RF, and ATCC 29212 was 0.03, 0.03, and 0.12, and the FIC of chloramphenicol was 0.125, 0.125, and 0.03. The total FICs of nisin and chloramphenicol were 0.155, 0.155, and 0.15, respectively. Therefore, the combination between nisin and penicillin or chloramphenicol was considered to have a synergetic effect against the three test *E. faecalis* strains.

### Bacterial Survival After Combined Treatment with Antibiotics and Nisin

**Figure 1:**
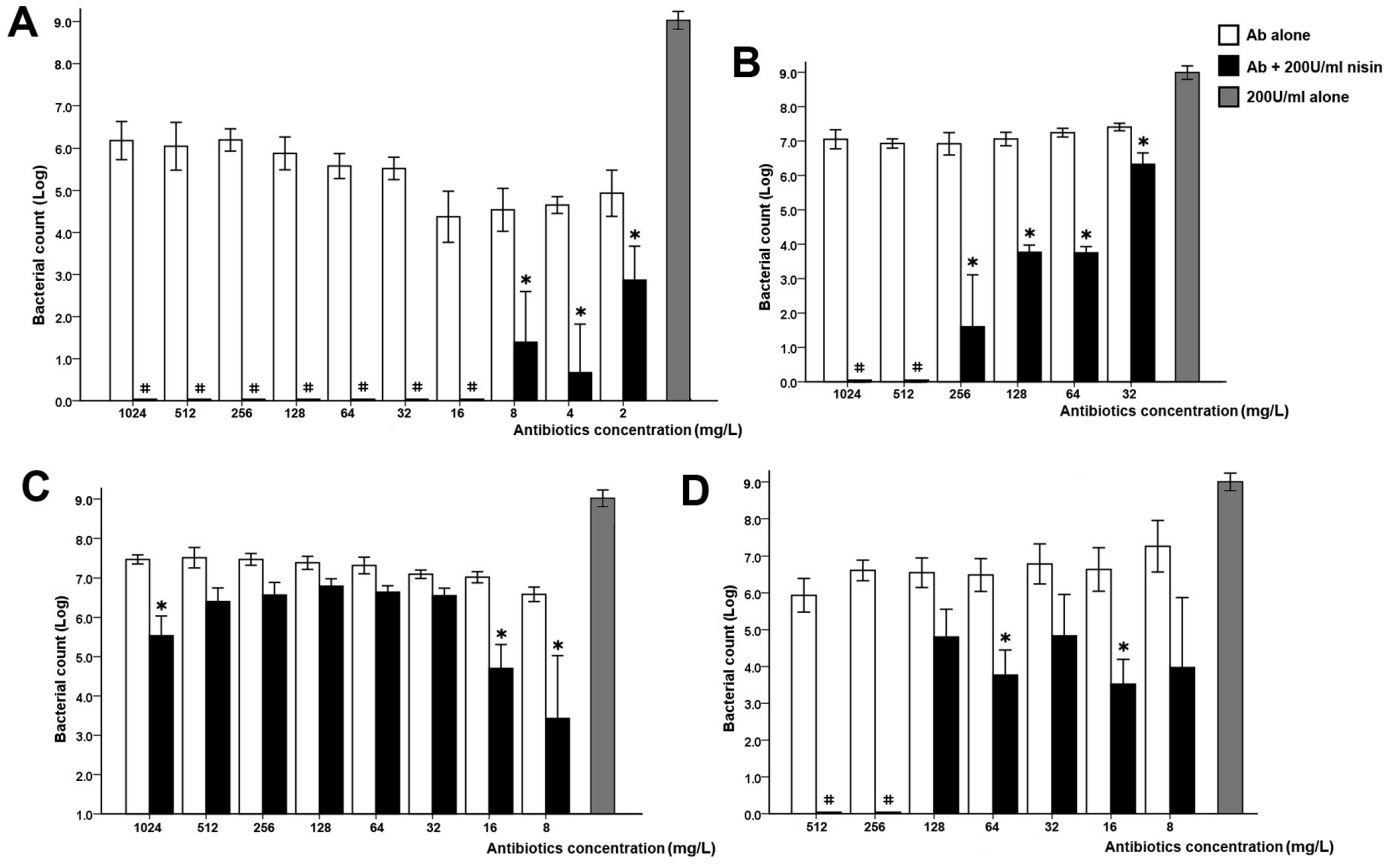
*E. faecalis* ATCC 29212 survival rates after treatment with different concentrations of penicillin (A), chloramphenicol (B), vancomycin (C), and linezolid (D) alone or in combination with 200 U/ml nisin. Raw *E. faecali*s colony counts were transformed to log10 values to normalize the data. “*” denotes a statistically significant difference between the survival rates of *E. faecalis* after exposure to the same concentration of antibiotics alone and antibiotics in combination with 200 U/ml nisin (*p*<0.05). “#” also denotes a statistically significant difference between groups with and without 200 U/ml nisin (*p*<0.05), as well as the fact that *E. faecali*s was completely killed by the antimicrobial agents.

The survival of *E. faecali*s ATCC 29212 was evaluated after 24 h of treatment with penicillin, vancomycin, chloramphenicol, or linezolid in combination with nisin (Fig. 1). In the control condition, 200 U/ml nisin alone did not inhibit *E. faecali*s growth, and *E. faecali*s grew to approximately 10^9^ CFU/ml. *E. faecali*s were not completely killed at 1024 mg/L penicillin, although its MBC was 16 mg/L. The *E. faecali*s survival rate did not decrease as the concentration of the four antibiotics increased. However, in the presence of 200 U/ml nisin, the bacterial survival rate significantly decreased, and statistically significant differences were observed between bacteria treated with or without nisin at the same concentrations of penicillin or chloramphenicol (*p*<0.05). In the presence of nisin, *E. faecali*s survival rates were shown to be significantly reduced; as the concentration of the two antibiotics increased, *E. faecali*s was completely killed at higher concentrations. Furthermore, 200 U/ml nisin was still able to improve the bactericidal effects of vancomycin and linezolid, although the benefits of the two antibiotics from nisin were not as substantial as those gained from the combination of nisin with penicillin or chloramphenicol.

### Morphological Changes of *E. faecalis*

**Figure 2:**
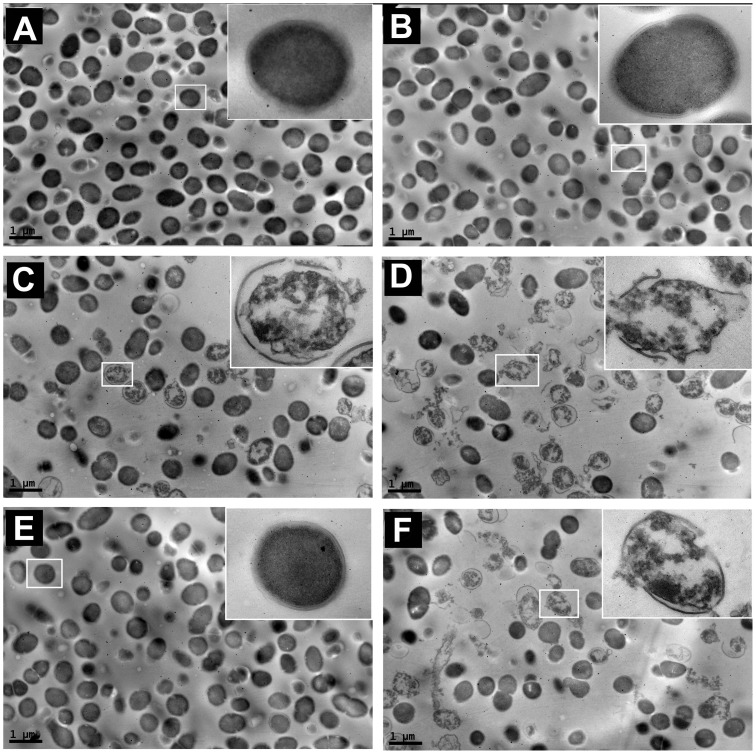
Morphological changes of *E. faecalis* ATCC 29212 observed by TEM after 12 hours of treatment with antibiotics in combination with nisin. (A) Control; (B) 1024 mg/L penicillin; (C) 2000 U/ml nisin; (D) 1024 mg/L penicillin and 2000 U/ml nisin; (E) 1024 mg/L chloramphenicol; and (F) 1024 mg/L chloramphenicol and 2000 U/ml nisin.

The TEM images of *E. faecali*s illustrated the morphological changes in the cells after antibiotic treatment. In this study, 2000 U/ml nisin was a suitable concentration that alone did not cause much of a morphological change in *E. faecali*s, but could enhance the damage induced by other antibiotics. The control *E. faecalis*, which was not treated with any drugs, exhibited normal sphericity, as a sign of morphological integrity (Fig. 2A). After 12 h of treatment with penicillin alone, the majority of *E. faecalis* still maintained their original shapes (Fig. 2B). The shapes of a few *E. faecalis* cells were destroyed after treatment with nisin alone (Fig. 2C). Under the combination of penicillin and nisin, many *E. faecalis* cells in the observation area lost their original morphology, instead showing distinct cellular disruption(Fig. 2D). Furthermore, upon treatment with chloramphenicol alone, *E. faecalis* cells did not show overt signs of cellular disruption, but a few cells were subjected to severe damage by the combination of chloramphenicol and nisin (Fig. 2E and 2F).

### Antibiofilm Assay

**Figure 3:**
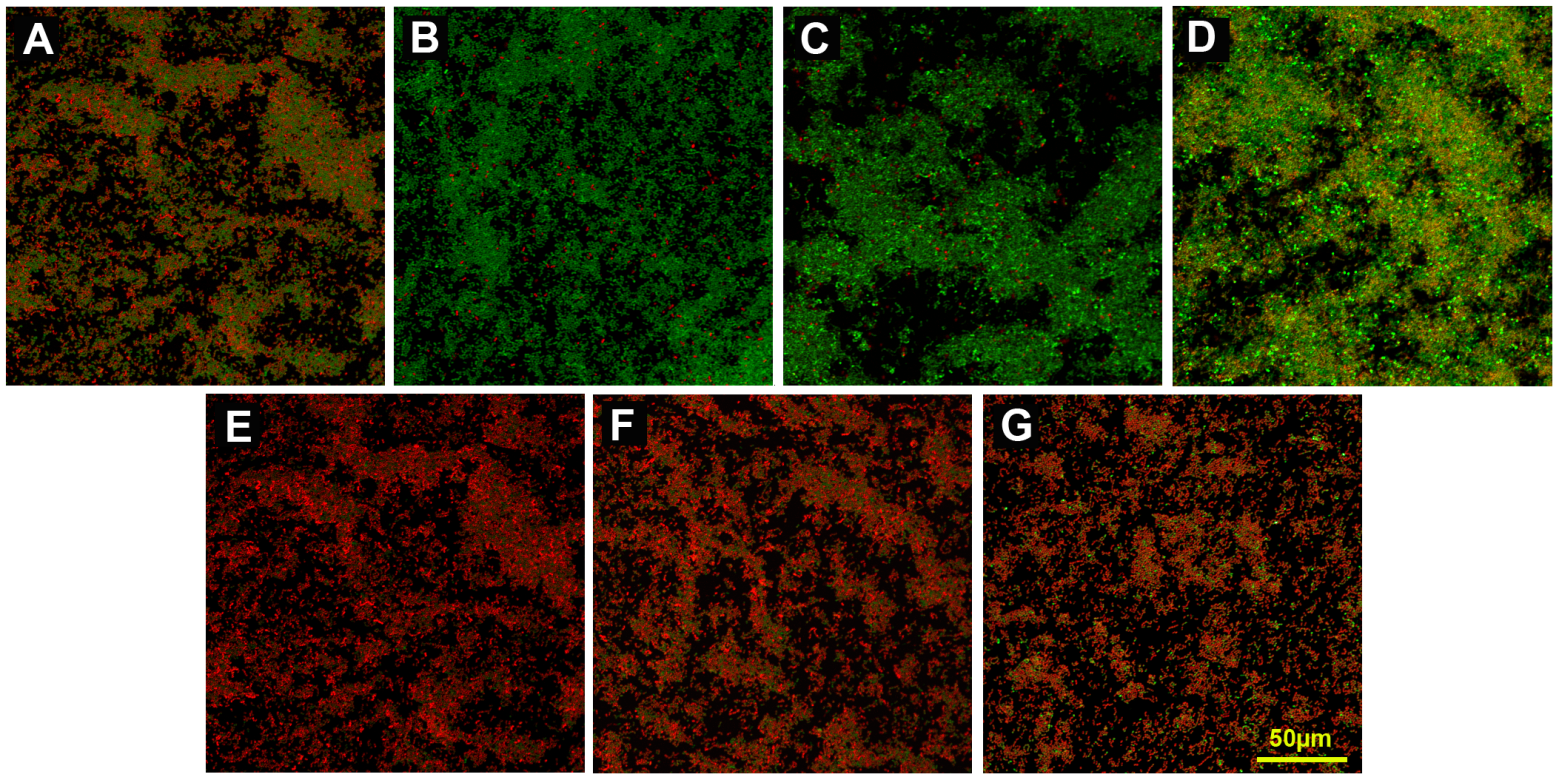
CLSM images showed the effects of antibiotics in combination with nisin on *E. faecalis* ATCC 29212 biofilms. (A) 512 mg/L penicillin; (B) 512 mg/L ciprofloxacin; (C) 512 mg/L chloramphenicol; (D) 400 U/ml nisin; (E) 512 mg/L penicillin and 400 U/ml nisin; (F) 512 mg/L ciprofloxacin and 400 U/ml nisin; (G) 512 mg/L chloramphenicol and 400 U/ml nisin. Bacteria with intact cell membranes are stained fluorescent green, whereas bacteria with damaged cell membranes are stained fluorescent red. All images were shown at 200× magnification and were collected using a Carl Zeiss CLSM.

**Figure 4:**
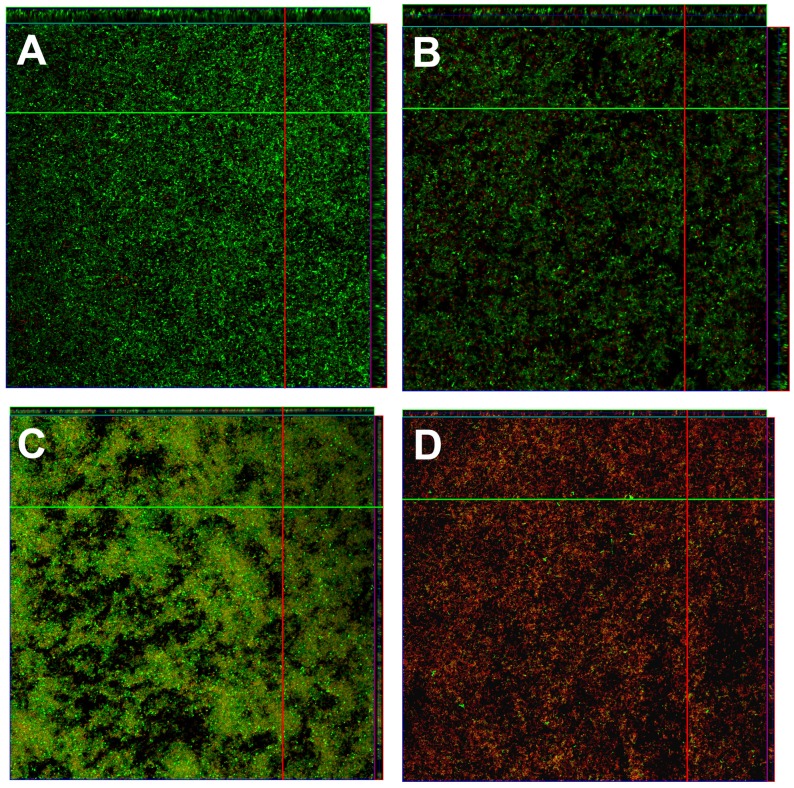
Twenty-four hours old *E. faecalis* ATCC 29212 biofilms were treated with (A) control biofilm with no treatment, (B) 512 mg/L penicillin alone, (C) 400 U/ml nisin alone, and (D) the combination of 512 mg/L penicillin and 400 U/ml nisin for 12 h. Their 3-dimensional images were scanned along the Z axis at different positions from bottom to top.

CLSM was used to examine the effects of penicillin, ciprofloxacin, or chloramphenicol in combination with nisin on *E. faecali*s biofilms (Fig. 3). In this study, 400 U/ml nisin was an ideal concentration that did not obviously destroy the *E. faecali*s biofilm, but could improve the testing of the antibiotics’ antibiofilm activity for *E. faecali*s. Viable cells were stained green and dead cells were stained red. *E. faecalis* biofilms exhibited extensive green areas, and a great deal of cells still survived after treatment with 512 mg/L penicillin, ciprofloxacin, chloramphenicol, or 400 U/ml nisin alone (Fig. 3A–3D). In particular, treatment with ciprofloxacin and chloramphenicol had little effect on the *E. faecali*s biofilm. However, treatment with a combination of penicillin, ciprofloxacin, or chloramphenicol and nisin led to a significant increase in the red areas of the *E. faecali*s biofilms, and the particular combination of penicillin and nisin was able to effectively kill the majority of cells in the biofilm (Fig. 3E–3G). Three-dimensional scanning of the control *E. faecalis* biofilm showed a green 3-dimensional space (Fig. 4A). After the *E. faecalis* biofilm was treated with nisin or penicillin alone, the whole layers almost appeared green, and small red areas were also found (Fig. 4B and 4C). However, *E. faecalis* from top to bottom in the biofilm were effectively killed by the combination of penicillin and nisin, and red dominated the 3-dimensional space (Fig. 4D).

## Discussion

*E*. *faecalis* is among the most antibiotic-resistant bacteria known at present. *E*. *faecalis* has the ability to quickly acquire and disseminate antibiotic resistance genes by pheromone signals produced within the genus and species as well as by other bacterial genera [21]. *E. faecalis* ATCC 29212 and OG1RF are generally used for survival and biofilm studies because they have been extensively used as a representative control strains for clinical and laboratory experiments [22], [23]. As *E. faecalis* has caused multiple antibiotic resistant infections, methods of effectively killing this drug-resistant pathogen have become key goals of microbiologists and drug development researchers. At present, vancomycin is considered a drug of last resort, and linezolid has also been introduced to treat severe infections with antibiotic-resistance Gram-positive bacteria [24], [25]. However, in the *in vitro* test for *E*. *faecalis*, not even these two potent antibiotics could effectively kill the three *E*. *faecalis* strains in this study. In contrast, the two conventional antibiotics penicillin and ampicillin exhibited better antibacterial activity and lower MIC and MBC values for penicillin and ampicillin than for vancomycin and linezolid. Similar results were found in a study by Weinstein *et al* [26]. Therefore, the results of the *in vitro* evaluation showed that penicillin and ampicillin may have better antibacterial effects on *E*. *faecalis* than vancomycin and linezolid.

The MBC has generally been defined as the lowest concentration of an antibiotic that kills >99.9% of the total bacteria [17], [27], [28], [29]. The MBC of penicillin against ATCC 29212 was 16 mg/L, and viable cells showed more than a 3-log10 reduction. However, in our determination, bacterial survival did not decrease, and even may have increased as the concentration of penicillin increased. Bacterial survival showed less than a 3-log10 reduction at >16 mg/L, so was 16 mg/L still considered the MBC? In an evaluation of the MBC of 18 test drugs, we found that no antibiotic completely killed *E*. *faecalis*, even at the high concentration of 1024 mg/L. These *in vitro* results indicated that *E*. *faecalis* is an antibiotic-resistant pathogen that is difficult to kill. The phenomenon that pathogens are relatively resistant to higher concentrations of some antibiotics while remaining susceptible to lower concentrations of antibiotics was first discovered by Eagle and Musselman in 1948 [30]. Nowadays, the phenomenon is often referred to as the “Eagle effect” and has been supported by additional studies [31], [32], [33], [34]. However, in our study the “Eagle effect” on *E. faecalis* did not take place with the addition of nisin, and *E. faecalis* was killed by the combination of the antimicrobial peptide nisin and many test antibiotics. A sub-MIC 200 U/ml concentration of nisin was used to clarify the antibacterial role of nisin in drug combinations. This concentration was significantly less than the MIC of nisin against the three *E*. *faecalis* strains and alone could not inhibit the bacterial growth, as the MICs of ATCC 29212, OG1RF, and strain E were found to be 1,000 U/ml. In the evaluation of bacterial survival rates, penicillin, chloramphenicol, and linezolid in combination with nisin could completely kill *E*. *faecalis*. This bactericidal effect was not due to the action of 200 U/ml nisin alone, but evidently nisin improves the bactericidal activities of these antibiotics. Especially with the addition of nisin, the low concentration of 16 mg/L penicillin resulted in complete bactericidal activity.

Many studies have indicated that nisin exerts its bactericidal activity by forming pores and inhibiting cell wall synthesis with a specific molecule, Lipid II, a principal component of the membranes of gram-positive bacteria [35], [36], [37], [38]. Nisin uses Lipid II as a “docking molecule” to form pores on the cell membrane surface in a targeted manner; at a nanomolar level, then, nisin is able to effectively kill bacteria [35], [39]. Therefore, 200 U/ml nisin is sufficient to form pores on the surface of bacteria and to facilitate the penetration of other antibiotic molecules into the microorganisms. In this way, antibiotics will better capture the antibacterial effects when their antibacterial action is occurring intracellularly. For example, macrolide antibiotics (Roxithromycin) binds irreversibly to a site on the 50S subunit of the bacterial ribosome and inhibits the translocation steps of protein synthesis [40]. Quinolone (ciprofloxacin) prevents bacterial DNA from unwinding and duplicating [41]. Aminoglycoside antibiotics (gentamicin, kanamycin, and streptomycin) work by binding to the bacterial 30S ribosomal subunit and inhibiting protein synthesis, and thereby compromise the structure of the bacterial cell wall [42], etc. The antibacterial activities of these antibiotics were obviously improved in the presence of a low concentration of nisin, 200 U/ml. This synergetic antibacterial mechanism involving the intracellular and cell membranes has been demonstrated in previous studies. The pores made by nisin allow more fluoride ions to enter *Streptococcus mutans* and for more doxycycline molecules to penetrate into *E*. *faecalis*; these actions result in the synergetic antibacterial activities of nisin and sodium fluoride, as well as of nisin and doxycycline [43], [44]. Furthermore, the study by Cottagnoud et al. showed that the cell wall disruption induced by vancomycin acts synergistically with gentamicin against penicillin-resistant pneumococci by increasing the intracellular penetration of gentamicin [45].

Nevertheless, 200 U/ml nisin was not sufficient to facilitate *E*. *faecalis* inhibition by sulfapyridine, metronidazol, or polymyxin. This may be due to the intrinsic resistance of *E*. *faecalis* to the three antibiotics; in our study, the MICs of the three antibiotics could not be detected. As a result, even if nisin helped a greater number of antibiotic molecules to access the bacteria, the antibiotics were not able to generate sufficient antibacterial activity.

In the evaluation of the antibacterial activity of the combination of penicillin and nisin, the results of the combination did not correspond with the mechanism of intracellular delivery by cell membrane disruption; however, the antibacterial activity of penicillin was still significantly strengthened in the presence of nisin. Penicillin is considered to bind to DD-transpeptidase, a penicillin-binding protein (PBP) that catalyzes the last step of peptidoglycan biosynthesis and thus prevents complete cell wall synthesis [46]. The mechanism of the cell wall disruption differs from that of nisin by Lipid II, an intermediate in the cell wall synthesis pathway. Therefore, the two antimicrobials attack the pathway differently, and are able to generate a greater disruption within the cell wall. This was also demonstrated by the TEM images. A majority of the cells were severely damaged by the dual attacks of penicillin and nisin and lost their original cell wall integrity. Similarly, this combined mechanism is seen in combinations of nisin and the cephalosporins (cefuroxime, cephazolin, ceftriaxone, and cefepime). However, *E*. *faecalis* cells appear more resistant to the dual action of vancomycin and nisin, and *E*. *faecalis* will still survive the challenge of the two antimicrobials in combination. Vancomycin decreases the accessibility of Lipid II by blocking the cell wall biosynthesis, and inhibites the membrane leakage activity of nisin against intact cells [35], [37]. Superior antibacterial activity is achieved by combining antimicrobials with different antibacterial mechanisms compared with a combination of antimicrobials with the same or similar mechanisms [47].

Bacterial biofilms generally become 10–1,000 times more resistant to the effects of antimicrobial agents than planktonic cells [48]. A majority of *E*. *faecalis* in the biofilm survived the challenges of penicillin, ciprofloxacin, and chloramphenicol, but nisin significantly improved the antibiofilm activities of the three antibiotics, with action taking place throughout many layers of the biofilm. Compared with some other bacteria, for example, *Streptococcus mutans*, *E*. *faecalis* form a biofilm that includes a substantial amount of eDNA but a low level of extracellular polysaccharides, leading to a low resistance to penetration by antimicrobial agents [49]. Antimicrobial molecules can easily enter this biofilm, and the high antibacterial activity of the antibiotics may play a key role in the inhibition of *E*. *faecalis* biofilms. Therefore, the potent antibacterial activity resulting from the combination of penicillin and nisin resulted in superior antibiofilm characteristics against *E*. *faecalis*. However, a CLSM image collected after treatment with penicillin and nisin includes some minor green areas, indicating that the potent antibacterial activity of the combination of these two antimicrobial agents had not yet completely killed all of the bacteria in the biofilm. Alternative strategies need to be explored in the future to improve the antibiofilm characteristics of these antibiotics.

These *in vitro* findings suggest that AMP nisin may significantly improve the antibacterial and antibiofilm activities of many antibiotics, and further, antibiotics in combination with nisin have considerable potential for use in the inhibition of drug-resistant pathogens.
